# Prognostic effect of factors involved in revised Tokuhashi score system for patients with spinal metastases: a systematic review and Meta-analysis

**DOI:** 10.1186/s12885-018-5139-2

**Published:** 2018-12-13

**Authors:** Xiong-gang Yang, Deng-xing Lun, Yong-cheng Hu, Yong-heng Liu, Feng Wang, Jiang-tao Feng, Kun-chi Hua, Li Yang, Hao Zhang, Ming-you Xu, Hao-ran Zhang

**Affiliations:** 1 Department of Bone Tumor of Tianjin Hospital, Tianjin, 300211 China; 2 Weifang People’s Hospital, Shandong, 261000 China; 3 Tianjin Medical university, Tianjin, 300070 China

**Keywords:** Spinal metastasis, Prognostic factor, Overall survival, Revised Tokuhashi score

## Abstract

**Background:**

Cancer patients’ survival time has obviously improved, with the development of systemic treatment techniques. However, the probability of metastases to the vertebrae has also been increased which makes some adverse effects on patients’ quality of life. The prediction of survival plays a key role in choosing therapeutic modality, and Tokuhashi Score was established as one of the most commonly used predictive systems for spinal metastases. Thus, this study was conducted to identify the prognostic effect of factors involved in revised Tokuhashi Score (RTS).

**Methods:**

Two investigators independently retrieved relevant literature on platforms of PubMed, Embase and Cochrane Library. We identified eligible studies through title/abstract and full-text perusing. Data was extracted including general information of studies, participants’ characteristics, therapeutic modality, overall survival and prognostic effect of factors. Hazard ratio (HR) for each factor was synthesized if available through fixed- or random-effect models as appropriate.

**Results:**

A total of 63 eligible studies with 10,411 participants were identified. Overall, cases with thyroid cancer had the highest survival rate, while the ones with non-small cell lung cancer and hepatocellular carcinoma lived for the shorted survival time. Performance status, bone metastasis, number of involved vertebrae, visceral metastasis, primary tumor and neurological status were regarded as significant predictors in 71.4, 40.0, 18.2, 63.4, 73.1 and 44.7% of the involved studies respectively. Thirty-eight articles were included in meta-analysis, and prognostic effects of five factors (apart from primary tumor) were analyzed. Factors were all proved to be significant except comparisons between KPS (Karnofsky Performance Status) 10–40 VS. 50–70 and single VS. multiple spinal metastases.

**Conclusion:**

All factors of RTS were significant on prognosis predicting and should be considered when choosing therapeutic modality for spinal metastases. What’s more, we believe that more accurate prognosis may be obtained after removal of the cut-offs for KPS 10–40 VS. 50–70 and single VS. multiple involved vertebrae.

**Electronic supplementary material:**

The online version of this article (10.1186/s12885-018-5139-2) contains supplementary material, which is available to authorized users.

## Background

With the improvements of systemic treatment techniques, cancer patients’ survival has obviously extended. However, the probability of metastases to the vertebrae has greatly increased, up to about 70%, which would make adverse effects on patients’ life quality [1, 2]. Patients suffered from spinal metastases usually have symptoms of intractable pain, neurological deficit and spinal instability, as the results of metastatic spinal cord compression (MSCC). In general, most of these patients are likely to benefit from aggressive surgery interventions while some are not if their life expectancies are extremely limited. Hence, for selecting of the optimal therapeutic modality, prognostic factors of the overall survival should be identified and taken into consideration.

Many studies have attempted to identify prognostic factors that predict survival of patients with spinal metastasis, and some handy scores have been established such as Tokuhashi [3, 4], Sioutos [5] and Tomita [6], Bauer [7], North [8] and Van der Linden [9]. Tokuhashi score is one of the most popularly used score systems for spinal metastases and most commonly reported in literature, which was originally established in 1990 and finally revised in 2005 [3, 4]. This score includes the following prognostic factors: performance status, bone metastases, number of involved vertebrae, visceral metastases, primary tumor type and neurological status. The type of primary tumor was scored between 0 and 5, while the other factors were scored between 0 and 2, which was added up to a maximum score of 15 (Table 1). According to this scoring system, if the total score is ranged 0–8, the predicted survival time will be less than 6 months and the conservative treatment or palliative surgery will be the optimal therapeutic modalities. For patients with a score of 12–15, the predicted survival time will be more than 12 months and more aggressive excisional surgery should be selected. And for patients with a score of 9–11, the predicted survival will be 6–12 months and palliative surgery or excisional surgery (a single vertebra was involved with no metastasis to major internal organs) will be recommended. The original authors have performed a validation study on the revised Tokuhashi Score (RTS) and shown an excellent accuracy as high as 87.9% between the predicted and actual survival. However, the accuracy of RTS in predicting the life expectancy for spinal metastases remain unsatisfying. Especially when cancer patients’ overall survival has been greatly improved because of more curative therapies (i.e. targeted therapy), the consistence and accuracy of RTS further decreased. As reported by Quraishi et al. [10], the prognostic criteria using RTS could only be moderately useful in predicting actual survival (66%). Pointillart et al. [11] also concluded from a prospective study that neither the original nor revised Tokuhashi scores were reliable in predicting survival in European population. The predictive value of the RTS was found to be less than 60%, and the prognostic effect of the factors showed conflicting results. For example, Tokuhashi [3, 4] included neurological deficit in the score, whereas Tomita [6], Bauer [7], North [8] and Van der Linden [9] did not.

**Table 1:** Revised Tokuhashi Score System for the Prognosis of Spinal Metastasis

Factors	Score
General condition (Karnofsky Performance Status, KPS)	
Poor (KPS 10–40)	0
Moderate (KPS 50–70)	1
Good (KPS 80–100)	2
Extraspinal bone metastases	
≥3	0
1–2	1
0	2
No. of metastases in the vertebral body	
≥3	0
2	1
1	2
Metastases to the major internal organs	
Unremovable	0
Removable	1
No metastases	2
Primary site of the cancer	
Lung, osteosarcoma, stomach, bladder, esophagus, pancreas	0
Liver, gallbladder, unidentified	1
Others	2
Kidney, uterus	3
Rectum	4
Thyroid, breast, prostate, carcinoid tumor	5
Neurological Status	
Complete (Frankel A, B)	0
Incomplete (Frankel C, D)	1
None (Frankel E)	2

Thus, the current study aimed to assess the effect of different parameters in RTS for predicting survival of patients with spinal metastases, and modify on the contents of RTS according to the significance of each parameter.

## Methods

### Data sources and searches

This review was conducted according to the guidelines outlined in Preferred Reporting Items for Systematic Reviews and Meta-analysis (PRISMA) statement. Two individual researchers (*Yang XG and Lun DX*) conducted platform searches on the PubMed, Embase and Cochrane Library. Literature retrieving was carried out through a combined searching of subject terms (“MeSH” on PubMed and “Emtree” on Embase) and free terms on PubMed and Embase, and through keywords searching on Cochrane Library. Searching strategies used on PubMed and Embase was presented in Additional file 1: Appendix 1. And the searching on Cochrane Library was conducted with the following keywords: “spinal metastasis; overall survival; prognostic factor”. Additionally, some else reference studies of relative articles and reviews were screened and hand-searched for possible inclusion.

### Inclusion and exclusion criteria for studies

Complete texts published between January 1997 and October 2017 (over the last two decades) with designs of cohort or case-control study approaching the survival and prognostic effect of factors included in RTS for patients with spinal metastases were included. The publication language was restricted in English but there were no limitations on the participants’ nationalities.

Studies would be excluded for the following reasons: (1) literature review, systematic review and/or meta analysis and letter to editors; (2) studies with less than 10 participants; (3) studies using repeated cohorts; (4) studies with high risk of bias according to the quality assessment; (5) duplicated studies.

### Study selection

After all duplicates were recognized and merged together by the software of EndNote X7 version 17.0 (Clarivate Analytics, Philadelphia, USA), the remained titles and abstracts were screened. Then, full texts of potentially relevant papers were obtained and assessed by full-text perusing for eligibility. The whole process of selection was strictly followed with the inclusion and exclusion criteria by two review authors (*Yang XG and Lun DX*) independently. Discrepancies in study selection between the two reviewers were handled by face-to-face discussion or judged by the third reviewer (*Liu YH*).

### Data extraction and quality assessment

Data was extracted by the two review authors pair independently and entered into a pre-built Microsoft Excel spreadsheet. Collected data included the following information: (1) characteristics of studies (title, author, publication year, country, study period, study design and quality of study), (2) participants’ characteristics (age, percentage of male, number of patients, number of patients with MSCC, primary tumor and spinal metastasis location); (3) therapeutic modality; (4) follow-up and overall survival; (5) prognostic effect of the factors and effect sizes for hazard ratio (HR) combined with their 95% confidence interval (95%CI) representing the prognostic value of factors included in RTS. We figured out causes of diversities on obtained information and resolved disagreements after discussion.

The Newcastle-Ottawa Scale (NOS) [12] was used for the assessment on risk of bias of the studies. This scale employs a 9 stars system that assesses three domains: patient selection, comparability of study groups and ascertainment of study outcome. Studies with a score of 8–9 stars have low risk of bias whereas scores of 6–7 mean medium bias risk and a score of 5 or less than 5 indicates a high chance of bias. Studies with a score of ≤5 stars would be excluded from this study.

### Quantitative data analysis

All recorded HRs and CI95% from eligible literature was pooled by an exploratory time-to-event meta-analysis with a random- or fixed-effect model as appropriate and heterogeneity was tested with I^2^ [13]. In case with significant heterogeneity (I^2^ > 50%), random-effect model would be employed, while fixed-effect model would be selected when presenting with excellent homogeneity (I^2^ < 50%). A test for the pooled effect sizes by Z test was performed and statistical significance was defined at a two-sided *P* value of less than 0.05. A sensitivity analysis would be performed when significant heterogeneity existing and studies causing instability would be removed. Publication bias was assessed with Begg’s and Egger’s regression asymmetry test (*p* < 0.050 and *p* < 0.100 were considered to be with significant publication bias respectively) [14]. In case with significant publication bias, a nonparametric trim and fill method will be performed to rectify the bias [15]. The whole process of meta-analysis was performed by Stata version 13.0 (StataCorp LLC, College Station, Texas, USA).

## Results

### Search result and study selection

**Fig. 1:**
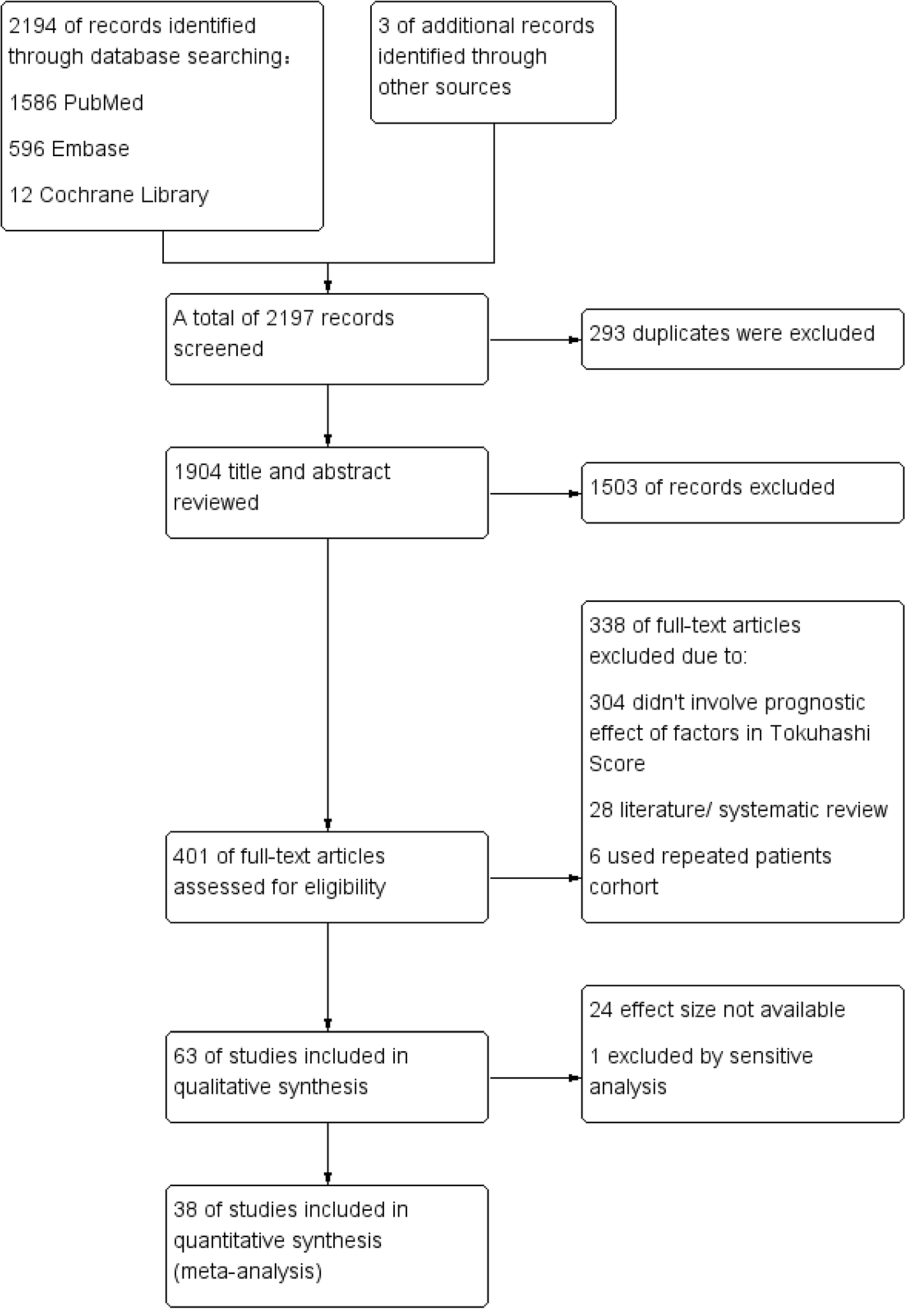
Flowchart of studies identification and selection

The flow chart of eligible literature selection was shown in Fig. 1. The initial searching on electronic platforms yielded a total of 2194 studies and another 3 articles were obtained by hand-searching. After exclusion of 293 duplicates, 1904 articles remained. Then by preliminary glancing over titles and abstracts and further perusing at full-texts, a number of 1503 and 338 articles were excluded respectively. The 338 full texts were excluded with the following reason: 304 studies didn^’^t involve prognostic effect of the factors involved in Tokuhashi Score; 28 studies were literature or systematic reviews; 3 studies of Lei [16–18] used repeated patients cohort, thus only the one [18] identified primary tumor histology as non-small cell lung cancer(NSCLC) was included; and another 4 studies of Rades [19–22] were also excluded for using repeated patients cohorts with other studies. Finally, 63 studies [6, 8, 9, 18, 24–72, 74–82] with 10,411 participants and 38 studies [8, 9, 18, 26, 28–38, 40, 41, 43, 44, 46, 47, 49, 51–53, 56, 58, 60, 63, 64, 66, 69, 71, 76, 78–81] with 7462 participants were included in the qualitative and quantitative synthesis respectively.

### General information of studies

**Fig. 2:**
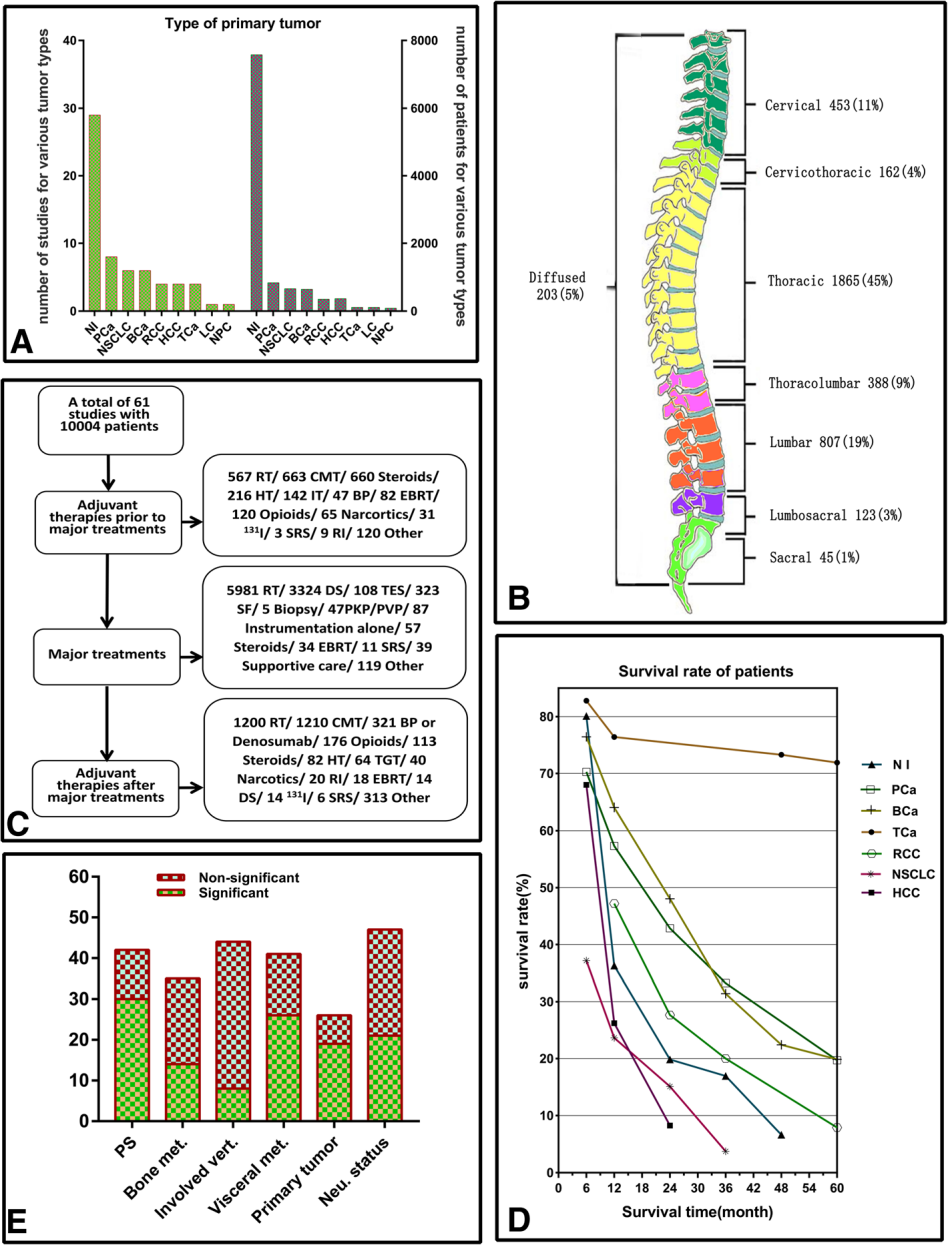
**a** Number of studies and patients for each type of primary tumor; **b** Distribution of spinal metastatic location; diffused patients include those presented with three or four sections of spinal metastases; **c** Therapeutic modalities provided for patients; **d** Overall survival rate for primary tumor; **e** Prognostic effect of factors included in revised Tokuhashi Score. (Note: NI = not identified; PCa = prostate cancer; NSCLC = non-small cell lung cancer; BCa = breast cancer; RCC = renal cell cancer; HCC = hepatocellular carcinoma; TCa = thyroid cancer; LC = lung cancer; NPC = nasopharyngeal carcinoma; RT = radiotherapy; CMT = chemotherapy; HT = hormonal therapy; IT = immunotherapy; BP = bisphosphonates; EBRT = external-beam radiation therapy; SRS = stereotactic radiosurgery; RI = radioisotopes; DS = decompression surgery; SF = spinal fusion; TGT = targeted therapy; PS = performance status; met. = metastases; Neu. = neurological)

Summary of individual study was listed in Table 2. Majority of the studies were of favourable quality assessed by NOS, with an average score of 7.8 ± 1.0 stars. None of the studies were excluded by quality assessment, which means no studies showed high risk of bias (NOS ≤ 5 stars). As for the delimitation, 57 and 4 studies were retrospective and prospective cohorts respectively, but only 1 each was case-control study and semi-retrospective cohort with a prospective manner on part of the information collection. Primary tumor histology was various among included studies, with 29 non-specified tumor type (7577 patients), 8 prostate cancer (842 patients), 6 non-small cell lung cancer (NSCLC, 667 patients), 6 breast cancer (648 patients), 4 renal cell cancer (355 patients), 4 hepatocellular carcinoma (371 patients), 4 thyroid cancer (110 patients) and 1 each for lung cancer (114 patients) and nasopharynx cancer (87 patients) (Fig. 2a).

**Table 2:** Summary of included studies

Author	Character of studies						Character of patients					
	Year	Study period	Study design	Country	Follow-up	NOS (Stars)	Primary tumor	Case	Case with MSCC	Male (%)	Age	Overall survival (median/mean)
van der Linden [9]	2005	1996–1998	retrospective cohort	Netherlands	≤32 m or until death	8	NI	342	12	53	mean: 66	median:7 m
Patchell [23]	2005	1992–2002	marched-pair study	USA	Median: surgery group: 3.4 m; radiation group: 3.1 m	9	NI	101	101	70	median: 60	NS
Chen [24]	2007	2000–2005	retrospective cohort	China	NS	8	NSCLC	31	31	61	mean: 61.4	median:8.8 m
Leithner [25]	2008	1998–2006	prospective+ retrospective cohort	Austria	≥12 m	7	NI	69	NS	54	mean: 60	median:14 m
Park [26]	2011	2001–2008	retrospective cohort	Korea	Mean: 25.8 m	8	NI	103	103	62	mean:54.6	median:10 m
Arrigo [27]	2011	1999–2009	retrospective cohort	USA	NS	9	NI	200	172	61	mean: 58.9	median:8 m
Rades [28]	2012	1992–2010	retrospective cohort	Germany	NS	8	NSCLC	356	356	74	median:64	NS
Crnalic [29]	2012	2003–2010	retrospective cohort	Sweden	NS	7	PCa	68	68	100	median:71	NS
Chong [30]	2012	2002–2010	retrospective cohort	Korea	NS	8	NI	105	105	69	mean:58.3	median:6 m
Rades [31]	2013	1992–2011	retrospective cohort	Germany	NS	8	NI	2029	2029	NS	NS	NS
Ju [32]	2013	2002–2011	retrospective cohort	USA	NS	8	PCa	27	27	100	median: 65	median:10.2 m
Bakker [33]	2014	2006–2013	retrospective cohort	Netherlands	NS	6	RCC	21	NS	NS	NS	median: 25 m
Bollen [34]	2014	2001–2010	retrospective cohort	Netherlands	Median: 6.6y	9	NI	1043	NS	52	mean:64.8	median:4.8 m
Vanek [35]	2015	2006–2012	retrospective cohort	Czech	NS	8	NI	166	166	NS	mean:62	median:16 m
Tang [36]	2015	2002–2013	retrospective cohort	China	Median: 13.5 m	9	NSCLC	116	116	65	median: 55	NS
Lei [18]	2015	2005–2015	retrospective cohort	China	Mean: 9.7 m	9	NSCLC	64	64	66	median:57	median:6.3 m
Chen [37]	2015	2000–2010	retrospective cohort	China	NS	8	NSCLC	50	50	68	mean: 61.6	median:7.5 m
Meng [38]	2016	2002–2012	retrospective cohort	China	NS	7	PCa	29	NS	100	median: 71	median: 44 m
Park [39]	2016	2010–2014	prospective cohort	Korea	NS	8	NSCLC	50	50	54	mean: 58.0	median:5.2 m
Huddart [40]	1997	1984–1992	retrospective cohort	UK	NS	8	PCa	69	69	100	NS	median: 3.8 m
North [8]	2005	NS	retrospective cohort	USA	NS	9	NI	61	NS	56	mean: 52.4	median:10 m
Williams [41]	2009	1993–2005	retrospective cohort	USA	NS	9	PCa	44	NS	100	median:68	median:5.4 m
Rades [42]	2012	1992–2010	retrospective cohort	Germany	NS	7	PCa	218	218	100	NS	NS
Crnalic [43]	2012	2003–2008	retrospective cohort	Sweden	Median: naïve: 26 m; refractory: 12 m	7	PCa	54	54	100	NS	NS
Lee [44]	2014	2005–2010	retrospective cohort	Korea	NS	7	NI	200	NS	59	mean: 59.9	mean: 10.8 m
Sellin [45]	2015	1993–2010	retrospective cohort	USA	NS	9	TCa	43	NS	60	NS	median:15.4 m
Drzymalski [46]	2010	1990–2009	retrospective cohort	USA	NS	8	PCa	333	77	100	median: 68	median:24 m
Tancioni [47]	2012	2004–2007	retrospective cohort	Italy	NS	9	NI	151	151	51	median: 62	median:14 m
Tatsui [48]	2014	1993–2007	retrospective cohort	USA	Median: 77.9 m	9	RCC	267	267	77	median:59.2	median:11.3 m
Petteys [49]	2016	2000–2011	retrospective cohort	USA	NS	8	RCC	30	NS	77	mean:57.6	median:11.4 m
Rades [50]	2016	NS	retrospective cohort	Germany	Median:6.5 m	7	TCa	14	14	29	median:70	NS
Kato [51]	2016	1984–2011	retrospective cohort	Japan	NS	7	TCa	32	NS	22	mean:60.6	median:6.4y
Sciubba [52]	2007	1993–2001	retrospective cohort	USA	Median: 13 m	9	BCa	87	NS	0	median: 53	median: 21 m
Walcott [53]	2011	2001–2009	retrospective cohort	USA	NS	7	BCa	15	15	0	median: 58	median: 34.2 m
Tancioni [54]	2011	2004–2009	retrospective cohort	Italy	Median:26 m	8	BCa	23	23	0	median:55	median:36 m
Zadnik [55]	2014	2002–2011	retrospective cohort	USA	Median:18.3 m	8	BCa	43	NS	0	median: 56	median:26.8 m
Ulmar [56]	2007	1984–2005	retrospective cohort	Germany	NS	6	RCC	37	20	84	median:64	mean:13.7 m
Jiang [57]	2014	1999–2013	retrospective cohort	China	Mean:42.7 m	7	TCa	21	NS	24	mean:62	NS
Oliveira [58]	2015	2010–2013	retrospective cohort	Brazil	mean: 13.8 m	7	NI	68	45	66	mean:62.2	NS
Kataoka [59]	2012	1990–2008	retrospective cohort	Japan	mean: 21 m	9	NI	143	NS	64	median:61	mean: 22 m
Aoude [60]	2016	2003–2012	retrospective cohort	Canada	NS	7	NI	126	NS	44	mean:59.2	mean:27 m
Bartels [61]	2007	1998–2005	retrospective cohort	Netherlands	NS	7	NI	219	185	58	mean:62.7	median:3 m
Lei [62]	2016	2005–2015	retrospective cohort	China	mean: 11.5 m	9	NI	206	206	51	median:56	median:7.3 m
Chang [63]	2001	1981–1997	retrospective cohort	China	NS	7	HCC	102	NS	93	mean:59.2	median:3 m
Chen [64]	2010	2001–2007	retrospective cohort	China	NS	7	HCC	41	NS	78	mean:53.2	mean:10.4 m
Choi [65]	2015	1992–2012	retrospective cohort	Korea	median:4.2 m	9	HCC	192	25	82	mean:56	median:4.5 m
Guo [66]	2003	1996–1998	retrospective cohort	USA	NS	6	NI	60	60	NS	NS	median:4.1 m
Moon [67]	2011	1987–2009	retrospective cohort	Korea	NS	6	NI	182	NS	61	median:56	median:8 m
Yang [68]	2012	2001–2009	retrospective cohort	Korea	NS	7	NI	217	NS	59	mean:55.5	median:6 m
Helweg-Larsen [69]	2000	a period of 3.5 years	prospective cohort	Denmark	≥11 m or until death	9	NI	153	153	51	NS	median:3.6 m
Kumar [70]	2014	2007–2011	retrospective cohort	Singapore	≥1y or until death	9	NPC	87	NS	78	median: 52	median:13 m
Mizumoto [71]	2008	2002–2006	retrospective cohort	Japan	≥1y or until death	9	NI	544	133	53	median:63	median:5.9 m
Ogihara [72]	2006	1993–2001	retrospective cohort	Japan	NS	7	LC	114	NS	61	mean:64.6	mean:6.2 m
Pointillart [11]	2011	2005–2007	prospective cohort	France	≥1y or until death	8	NI	142	NS	57	mean:61.8	median:5 m
Rades [73]	2006	1992–2003	retrospective cohort	Germany	NS	7	BCa	335	335	0	NS	median:20 m
Switlyk [76]	2015	2007–2008	retrospective cohort	Norway	NS	7	NI	173	47	56	median:65	median:8.2 m
Tao [74]	2004	1992–2002	retrospective cohort	China	≥6 m	9	NI	63	NS	59	mean:52	mean:6 m
Tomita [6]	2001	1987–1991	retrospective cohort	Japan	until 1992	7	NI	67	NS	46	mean:56.3	NS
Weber [76]	2013	NS	retrospective cohort	Germany	NS	6	BCa	145	145	0	median:63	NS
Yamashita [77]	2011	2006–2008	prospective cohort	USA	≥1y	9	NI	85	NS	52	mean:60.3	median:11.6 m
Yeung [78]	2014	2000–2010	retrospective cohort	China	≥1y	9	NI	128	128	71	mean:60.2; median:59	mean:7.6 m
Zhang [79]	2013	2003–2011	retrospective cohort	China	mean: 15.7 m	9	HCC	36	NS	89	mean:49.9	NS
Enkaoua [80]	1997	NS	retrospective cohort	France	NS	6	NI	71	NS	51	mean: 59.8	NS

Note: BCa = breast cancer; HCC = hepatocellular carcinoma; LC = lung cancer; MSCC = metastatic spinal cord compression; NI = not identified; NOS = The Newcastle-Ottawa Scale; NPC = nasopharyngeal carcinoma; NS = not specified; NSCLC = non-small cell lung cancer; OS% = percentage of overall survival; PCa = prostate cancer; RCC = renal cell cancer; RT = radiotherapy; SUR = surgery; TCa = thyroid cancer

### Participants’ characteristics

Of the 63 studies eligible for inclusion, 36 reported number of patients with MSCC before treatment, which added up to 5820 in 7212 patients (80.7%). Apart from 14 studies for prostate and breast cancer, 45 studies reported percentage of gender, with 4169 (59.5%) males and 2836 (40.5%) females included. An overall mean age of 4564 patients involved in the 31 studies was 61.9 years. Regarding the location of metastases, data was available in 36 studies containing 4046 patients, and maximum number of patients developed thoracic metastasis, followed by lumbar, cervical, thoracolumbar, diffused, cervicothoracic, lumbosarcral and sacrum metastasis (Fig. 2b).

### Therapeutic modality

Modality of therapy was available in 61 articles containing 10,004 patients (Fig. 2c). Patients predominantly received surgery or radiotherapy as major treatments. Surgery types mainly included 3324 decompression surgery with/without instrumented procedures, 108 total en bloc spondylectomy, 323 spinal fusion. Radiotherapy was performed in 5981 patients as major treatment. Other treatments, such as adjuvant therapies, radiotherapy, chemotherapy, targeted therapy, immunotherapy, bisphosphonates, were provided alone or with various combination prior to or after major procedures.

### Follow-up and overall survival

Data of follow-up was available in 27 studies, and 7 of them were followed for more than one year or until death. 7 were followed for an average period ranged 9.7–42.7 months and 10 were followed for a median period ranged 3.1–79.2 months. After treatment, the average survival time was ranged 6–27 months, and median survival time was ranged 3–77 months as reported in 8 and 42 studies respectively. Survival rates at 6, 12, 24, 36, 48 and 60 months for various types of primary tumors were calculated and presented in Fig. 2d. Overall, thyroid cancer had the highest survival rate, followed by prostate cancer/ breast cancer, renal cell cancer and mixed cancer, and non-small cell lung cancer and hepatocellular carcinoma lived for the shorted life span.

### Qualitative data summary on prognostic factors

Numbers of studies that showe significance and non-significance for each prognostic factor are presented in Fig. 2e. Performance status was analyzed in 42 articles and 30 (71.4%) supported it as a significant factor. Prediction value of bone metastasis was involved in 35 studies, and 14 (40.0%) reported statistical significance. Number of involved vertebrae was analyzed in 44 studies, and 8 (18.2%) studies drew significant conclusions. As for visceral metastasis, 26 (63.4%) studies regarded it as a significant predictor in 41 involved studies. Totally, 26 studies analyzed the influence of primary tumor on survival, and 19 (73.1%) of them were ofstatistical sig nificance. Neurological status was involved in 47 studies and 21 (44.7%) were statistically significant.

### Quantitative data synthesis

**Fig. 3:**
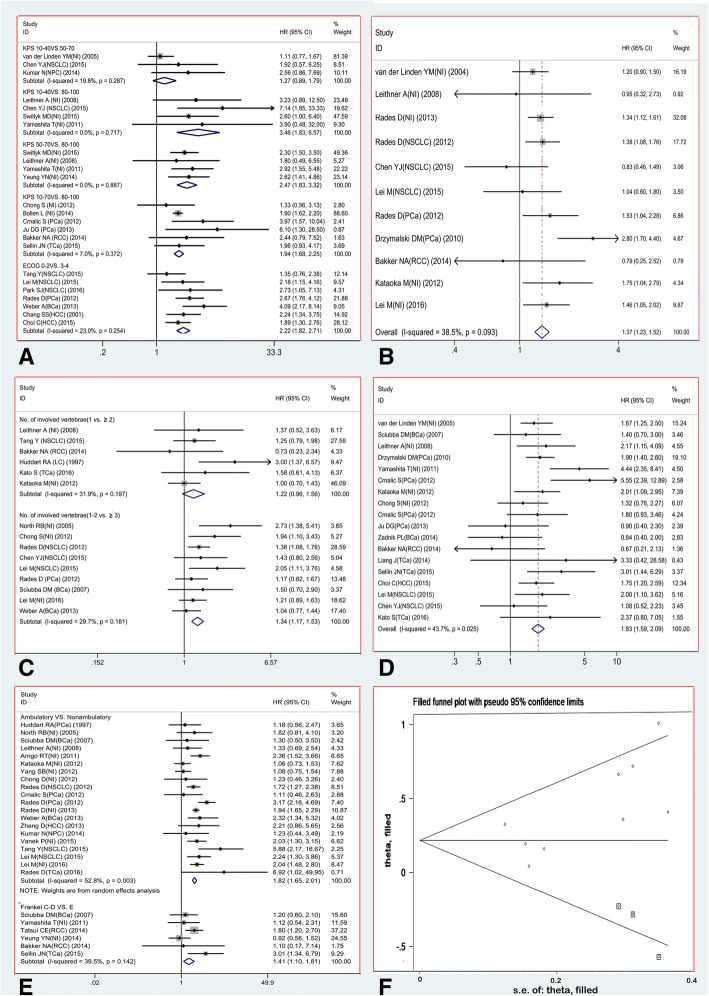
**a** Forest plots for effect size of performance status (KPS/ ECOG); **b** Forest plot for effect size of arising of other bone metastasis; **c** Forest plot for effect size of number of involved vertebrae; **d** Forest plot for effect size of arising of visceral metastasis; **e** Forest plot for effect size of neurological status; **f** Funnel plot after 3 studies were filled by a nonparametric trim and fill method (the diamonds represent studies which were filled)

Prognostic effects of five factors (primary tumor type was not included for lack of homogeneous comparison between groups) were identified. The results of meta-analyses are presented in Table 3. As shown in these results, patients with ‘severe’ disability (KPS 10–40) and ‘moderate’ disability (KPS 50–70) have similar survival rates (HR = 1.27, CI 95% 0.89–1.79, *P* = 0.186) and both groups are worse than patients with no to mild disability (KPS 80–100) (Fig. 3a). And patients with 3 or more involved vertebrae have worse survival than patients with 1–2 involved vertebrae, while patients with single and multiple involved vertebrae have similar survival rates (HR = 1.22, CI 95% 0.96–1.56, *P* = 0.102) (Fig. 3c). All the other comparisons between various groups of patients for the five prognostic factors were proved to be significant (Fig. 3a-e). All the meta-analyses were performed with a fixed-effect model except comparison between ambulation and non-ambulation (I^2^ = 52.8%). Egger’s test for number of involved vertebrae (1–2 VS. ≥3) presented a significant publication bias (*P* = 0.046) and a nonparametric trim and fill method was performed to rectify the detected publication bias (Fig. 3f). Pooled effect size of HR was 1.24 (CI 95% 1.10–1.40, *P* = 0.001) after 3 studies were filled.

**Table 3:** Results of quantitative meta-analyses

Prognostic factor	No. of studies	No. of patients	Pooled effect size(HR)	CI 95%	I^2^ (%)	Effect model	Z test (*P* value)	Excluded studies by sensitivity analysis	Publication bias (*P* value)	
									Begg’s	Egger’s
KPS(10-40VS.50–70) [9, 38, 71]	3	479	1.27	(0.89, 1.79)	19.8	Fixed	0.186^a^	0	1.000	0.188
KPS(10-40VS.80–100) [11, 26, 38, 76]	4	377	3.46	(1.83, 6.57)	0.0	Fixed	< 0.001	3 [9, 71, 79]	0.308	0.404
KPS(50-70VS.80–100) [26, 75, 78, 79]	4	455	2.47	(1.83, 3.32)	0.0	Fixed	< 0.001	0	1.000	0.834
KPS(10-70VS.80–100) [30, 31, 32–35, 46]	6	1307	1.94	(1.68, 2.25)	7.0	Fixed	< 0.001	0	0.133	0.214
ECOG(1-2VS.3–4) [19, 37, 40, 43, 64, 66, 75]	7	887	2.22	(1.82, 2.71)	23.0	Fixed	< 0.001	4 [29, 32, 60, 72]	0.548	0.345
Extraspinal bone metastases [9, 19, 26, 29, 32, 34, 38, 43, 47, 60, 70]	11	3831	1.37	(1.23, 1.52)	38.5	Fixed	< 0.001	0	0.755	0.819
No. of involved vertebrae (≥2VS.1) [26, 34, 37, 41, 52, 60]	6	450	1.22	(0.96, 1.56)	31.9	Fixed	0.102^a^	0	1.000	0.434
No. of involved vertebrae (≥3VS.1–2) [8, 19, 29, 31, 38, 43, 53, 63, 75]	9	1292	1.34	(1.17, 1.53)	29.7	Fixed	< 0.001	0	0.118	0.046^b^
Visceral metastases [9, 19, 26, 30, 31, 33, 34, 38, 44, 46, 47, 52, 53, 56, 58, 60, 66, 76]	18	1779	1.83	(1.59, 2.09)	43.9	Fixed	< 0.001	7 [28, 29, 32, 33, 35, 43, 74, 72]	0.880	0.969
Ambulatory status [8, 19, 26, 28–32, 36, 37, 41, 43, 51, 53, 60, 63, 69, 71, 75]	20	4456	1.80	(1.52, 2.13)	52.8	Random	< 0.001	0	0.922	0.953
Frankel (C-D VS. E) [34, 46, 49, 53, 76]	6	631	1.41	(1.10, 1.81)	39.5	Fixed	0.006	0	0.707	0.967

Note: ^a^Pooled effect sizes were considered to be non-significant statistically (*P* value was more than 0.05 by Z test); ^b^A significant publication bias was existed according to Egger’s test and the nonparametric trim and fill method was performed to rectify the bias

According to these results, remodifications on the cut-off of KPS and number of involved vertebrae were conducted for the RTS, and a remodified version of RTS is shown in Table 4. Patients with KPS 10–40/50–70 and patients with single/double involved vertebrae were merged together and the total score of the RTS was not changed which was added up to 15.

**Table 4:** A remodified Version of Revised Tokuhashi Score System

Factors	Score
General condition (Karnofsky Performance Status, KPS)^a^	
Poor and moderate (KPS 10–70)	0
Good (KPS 80–100)	2
Extraspinal bone metastases	
≥3	0
1–2	1
0	2
No. of metastases in the vertebral body^b^	
≥2	0
1	2
Metastases to the major internal organs	
Unremovable	0
Removable	1
No metastases	2
Primary site of the cancer	
Lung, osteosarcoma, stomach, bladder, esophagus, pancreas	0
Liver, gallbladder, unidentified	1
Others	2
Kidney, uterus	3
Rectum	4
Thyroid, breast, prostate, carcinoid tumor	5
Neurological Status	
Complete (Frankel A, B)	0
Incomplete (Frankel C, D)	1
None (Frankel E)	2

Note: This remodified version of RTS was raised according to results in the meta-analyses and remodifications on the cut-off of KPS (^a^) and number of involved vertebrae (^b^) were conducted for the scoring system. The patients with KPS 10–40/ 50–70 and patients with single/double involved vertebrae were merged together

## Discussion

The primary aim of the treatment on spinal metastasis is to attain the optimal relief on symptoms of MSCC (e.g. intractable pain and neurological deficit), restore or maintain of spinal stability and improving the quality of life by various individualized therapeutic options. A number of prognostic scoring systems have been established to assist clinicians in predicting prognosis, such as Tokuhashi [3, 4], Tomita [6] and Enkaoua [82]. To achieve the optimal remission of symptoms, surgeons must consider patients’ life expectancy. However, most of the scores present sources of bias in patient selection and involve conflicting factors. According to RTS, performance status, bone metastasis, number of involved vertebrae, visceral metastasis, primary tumor and spinal cord palsy are significant to predict patients’ overall survival [3, 4]. Current study identified the role of factors included in RTS in predicting overall survival in patients with spinal metastases.

### Prognostic effect of factors

#### General condition

Rades [43] compared overall survival of patients with Eastern Cooperative Oncology Group (ECOG) performance status 1–2 and 3–4, and the former group was presented with a significant higher survival. Van der Linden [9] and Bartels [62] also included performance status in their prognostic scores. Generally, patients with better performance status could tolerate more invasive therapeutic modalities, which would extend patients’ survival. However, some other studies did not considered performance status as a significant predictor. Leithner [26] supposed some other factors, such as arising of visceral metastasis and sever neurological deficit, would also make patients debilitated, and further decreased patients’ performance status, but these patients might be favourable in otherwise general condition to tolerate invasive therapy. In current study, performance status was identified to be a significant predictor for all except comparison between KPS 10–40 and 50–70 (*P* = 0.186). Thus, in general, performance status could be identified to be a reliable predictor. Similar to the results of the previous studies [9, 38, 71], we thought that the cut-off of KPS should not included KPS 10–40/50–70 as patients were both too debilitated to be cured from invasive therapies.

#### Extraspinal bone metastases and number of involved vertebrae

Rades [32] found that bone metastasis was significant in predicting prognosis of patients treated with radiotherapy. In study of Chong [31], patients with ≤2 column involved had a significant longer overall survival than the ones with > 2 column involved. Generally, the two factors were often related to biological behaviour of invasion, spread and proliferation, which indicates advanced stages of cancer. In addition, added number of involved vertebrae would increase the difficulty of treatment and probability of occurrence of complications. Meanwhile, many studies presented non-significant results on prognosis effect of the two factors, such as van der Linden [9]. And Tomita Score adopted bone metastasis but not number of spinal metastases [6]. In current study, extraspinal bone metastases and number of involved vertebrae (≥3 VS. 1–2) were confirmed to be significant factors, but number of involved vertebrae (multiple VS. single) was of non-significance. Overall, we think that the two factors are reliable but the cut-off of number of involved vertebrae should not included single/ multiple spinal metastases, and use of > 1 vertebrae as cutoff is less effective for predicting survival than use of > 2 vertebrae.

#### Visceral metastases

In scores of Tomita [6], van der Linden [9] and Enkaoua [82], visceral metastasis is included as a predictor. Rades [29] found that not only arising of visceral metastases with ≥2 sites had a poorer prognosis than arising of 0–1 site, patients with and without metastasis also had a diverse survival. Generally, visceral metastases is considered as a significant factor due to 3 reasons: (1) it is often related to an advanced stage of cancer; (2) it may increase number of complications; (3) it deliver more metastatic burden to patients than spinal metastasis. However, Bollen [35] found that visceral metastasis was not a significant factor for all but patients with favourable primary tumor types, and patients with moderate and unfavourable profile of primary tumors were of very poor prognosis that prognostic effects of visceral metastases were weakened. Regardless of existed controversies, our meta-analysis identified visceral metastases as a significant predictor (*P* < 0.001).

#### Histology of primary tumor

As reported by Arrigo [28], primary tumor was a robust predictor in spinal metastasis. Yeung [80] also found that primary tumor types by RTS was a significant predictor overall. Nevertheless, a minority of studies presented a non-significance on the prognostic effect of primary tumor [19, 31, 36]. As reported in study of Lee [45], discrepancy of survival among different primary tumors were not significant. And they insisted that it’s due to some advanced adjuvant therapeutic modalities that make patients with primary tumor of high malignancy lived a longer survival. In current study, we figured that thyroid cancer had the highest survival rate, followed by prostate/ breast cancer, renal cell cancer and mixed cancer, and non-small cell lung cancer and hepatocellular carcinoma lived for the shorted life span, which was in accordance with RTS [4].

#### Neurological status

Sioutos [5] and Enkaoua [82] included neurological deficit in their scores. Rades [22] and Tang [37] also accepted ambulatory status as a significant factor, since patients with neurological deficit might become too deteriorated to tolerate more aggressive surgical procedures and adjuvant therapies, and more severe complications would arise among paraplegic patients. However, there were also many studies that did not adopt neurological status as a predictor based on their cohorts such as Tomita Score [6]. They insisted that neurological deficit could be improved through appropriate treatment, which would bring about a longer survival. Van der Linden [9] speculated that symptom of myeloplegia could just reflect the location and volume of lesions but not the biological behaviour. In current study, both of ambulatory status and arising of neurological deficit before treatment were confirmed to be significant, which was in accordance with RTS [4].

#### Remodification on the revised Tokuhashi score

Tokuhashi Scoring was developed for the preoperative evaluation on the prognosis of metastatic spinal tumors and has been used clinically with minor revisions [3, 4]. For the revised score, consistency rate between the predicted prognosis from the criteria of the total scores and the actual survival was proved to be as high as 86.4% in the 118 patients evaluated prospectively after 1998 [4]. Yamashita [79] identified the relation between the revised Tokuhashi score and actual survival of 85 patients and found that actual survival matched the predicted survival in 67 (79%) of 85 patients. Thus, RTS was found to be very effective to predict survival. Nevertheless, some studies identified the RTS as a less predictive and practicable prognostic system [10, 83]. Gakhar [83] found that RTS was only significantly accurate in group of patients with expected survival of more than 12 months but not in groups with less than 1 months or between 6 to 12 months. According to current study, in general, factors of RTS were all valuable in predicting survival as many studies had verified [65, 71]. While more accurate prognosis may be obtained if remodifications were made on the cut-off of KPS and number of involved vertebrae were conducted for the scoring system in future. Considering the results of quantitative pooling, we thought that patients with KPS 10–40/50–70 and patients with single/double involved vertebrae should be merged together.

Though RTS was proved to be practicable and accurate for predicting the life expectancy of patients with spinal metastasis in plenty of former studies as well as the current study, it was also limited since it had only analyzed the prognostic effect of preoperative characteristics. The RTS has been used for a long term after it was first established in 1990 and revised in 2005. But to our knowledge, the significant predictors for spinal metastasis have been changed over the time, especially after some effective adjuvant interventions, such as target or chemical therapies have been applied to the clinical treatment. The patients’ life expectancy have been obviously altered in some specific tumor types in the recent years. For instance, after the introduction of the anti-VEGF antibody Bevacizumab combined with a Cisplatin-containing regimen was used in nonsquamous NSCLC, and the patients’ progression-free survival was significantly improved [82]. In the study of Horn et al., [83] it was also demonstrated that Bevacizumab (more than 14 months) significantly improved the overall survival of patients with adenocarcinoma compared standard therapy (10 months). Hence, apart from the factors that has been involved in the RTS, we propose establishing new scores or new revisions on RTS in the future to sufficiently consider the effect of modern therapeutic modalities, which would further increase the accuracy and prognostic capacity on predicting the patients’ survival.

#### Limitations of this study

Our study nonetheless has limitations. Firstly, primary articles included were published with design of retrospective cohorts dominantly, and only an average value of 7.8 ± 1.0 stars for NOS was presented which would cause some potential bias. It may be due to few prospective cohort studies have been carried out till now. Anyhow, majority of studies were of an acceptable quality and none was showed to be with high risk of bias (NOS ≤ 5 stars). Secondly, the studies included in this work lacked information on either one or more RTS parameter(s) as few studies had completely contained and reported the data about each of the parameter, which would lead to an inevitable bias. What’s more, current study could only evaluate and verify the prognostic effects of the factors in Tokuhashi Score, but we did not assess the accuracy of predicted survival time for patients with various levels of Tokuhashi scores.

## Conclusion

Factors included in RTS were all significant on prognostic predicting for patients with spinal metastasis and should be considered when choosing the appropriate treatment modality. What’s more, we believe that more accurate prognosis may be obtained by merging patients with KPS 10–40/KPS 50–70 and patients with single/ double involved vertebrae together. Using the modified RTS, patients present with a low score are predicted to live a short period and some palliative therapies should be applied, while patients should be treated with invasive procedures when present with a high RTS score. Additionally, we suggest that more sufficiently considering on the effect of modern therapies is necessary for developing new scores in the future, as adjuvant interventions have significantly altered the patients’ life expectancy in the recent years.

## Additional file

Additional file 1: **Appendix 1.** Searching strategies used for the literature retrieving. (DOCX 13 kb)

## Abbreviations

CI: confidence interval
ECOG: Eastern Cooperative Oncology Group
HR: hazard ratio
MSCC: metastatic spinal cord compression
NOS: the Newcastle-Ottawa Scale
PS: performance status
RTS: revised Tokuhashi Score
