# Supplementary material for: Prognostic effect of factors involved in revised Tokuhashi score system for patients with spinal metastases: a systematic review and Meta-analysis
Source: BMC Cancer. 2018 Dec 13;18:1248. doi: 10.1186/s12885-018-5139-2 (PMC6293585; doi:10.1186/s12885-018-5139-2)
Supplement: Supplementary file 1 — Appendix 1. Searching strategies used for the literature retrieving. (DOCX 13 kb) [file 12885_2018_5139_MOESM1_ESM.docx]

The searching on Pubmed was performed using the following strategy:

*#1 (Spinal Metastasis[MeSH]) OR (Spinal Metastases) OR (Metastases, Spinal) OR (Metastasis, Spinal) OR (Metastatic Spinal Cord Compression) OR MSCC OR (Spinal Epidural Metastases) OR SEM OR (Vertebral Metastases) OR (Vertebral Metastasis) OR (Metastatic Spinal Tumors)*

*#2 (Prognosis[MeSH]) OR Prognoses OR (Prognostic Factors) OR (Factor, Prognostic) OR (Factors, Prognostic) OR (Prognostic Factor) OR (Prognostic Effect)*

*#3 (Survival[MeSH]) OR (Overall Survival) OR (Post-operative Survival) OR (Post-treatment Survival)*

*#4 Filters: Publication date from 1997/01/01 to 2017/10/01*

*#5 #1 AND #2 AND #3 AND #4*

The searching on Embase was performed using the following strategy:

*#1 'Spine Metastasis'/exp OR ‘Spinal Metastasis':ab,ti OR ‘Spinal Metastases':ab,ti OR ‘Metastases, Spinal':ab,ti OR ‘Metastasis, Spinal':ab,ti OR ‘Metastatic Spinal Cord Compression':ab,ti OR ‘MSCC':ab,ti OR ‘Spinal Epidural Metastases':ab,ti OR ‘SEM':ab,ti OR ‘Vertebral Metastases':ab,ti OR ‘Vertebral Metastasis':ab,ti OR ‘Metastatic Spinal Tumors':ab,ti*

*#2 'Prognostic Factor'/exp OR ‘Prognosis':ab,ti OR ‘Prognoses':ab,ti OR ‘Prognostic Factors':ab,ti OR ‘Factor, Prognostic':ab,ti OR ‘Factors, Prognostic':ab,ti OR ‘prognostic Effect':ab,ti OR ‘Prognostic Value':ab,ti OR ‘Prognostic Model':ab,ti*

*#3 'Overall Survival'/exp OR ‘Survival':ab,ti OR ‘Post-operative Survival':ab,ti OR ‘Post-treatment Survival':ab,ti*

*#4 [1997-2017]/py*

*#5 #1 AND #2 AND #3 AND #4*
